# T1 at 1.5T and 3T compared with conventional T2* at 1.5T for cardiac siderosis

**DOI:** 10.1186/s12968-015-0207-0

**Published:** 2015-11-24

**Authors:** Mohammed H. Alam, Dominique Auger, Gillian C. Smith, Taigang He, Vassilis Vassiliou, A. John Baksi, Rick Wage, Peter Drivas, Yanqiu Feng, David N. Firmin, Dudley J. Pennell

**Affiliations:** NIHR Cardiovascular Biomedical Research Unit, Royal Brompton Hospital, Sydney Street, London, SW3 6NP UK; Imperial College London, London, UK; St George’s, University of London, London, UK; School of Biomedical Engineering, Southern Medical University, Guangzhou, China

**Keywords:** Cardiac siderosis, 3 Tesla, T1 mapping, MOLLI, T2*, Cardiovascular magnetic resonance

## Abstract

**Background:**

Myocardial black blood (BB) T2* relaxometry at 1.5T provides robust, reproducible and calibrated non-invasive assessment of cardiac iron burden. In vitro data has shown that like T2*, novel native Modified Look-Locker Inversion recovery (MOLLI) T1 shortens with increasing tissue iron. The relative merits of T1 and T2* are largely unexplored. We compared the established 1.5T BB T2* technique against native T1 values at 1.5T and 3T in iron overload patients and in normal volunteers.

**Methods:**

A total of 73 subjects (42 male) were recruited, comprising 20 healthy volunteers (controls) and 53 patients (thalassemia major 22, sickle cell disease 9, hereditary hemochromatosis 9, other iron overload conditions 13). Single mid-ventricular short axis slices were acquired for BB T2* at 1.5T and MOLLI T1 quantification at 1.5T and 3T.

**Results:**

In healthy volunteers, median T1 was 1014 ms (full range 939–1059 ms) at 1.5T and modestly increased to 1165ms (full range 1056–1224 ms) at 3T. All patients with significant cardiac iron overload (1.5T T2* values <20 ms) had T1 values <939 ms at 1.5T, and <1056 ms at 3T. Associations between T2* and T1 were found to be moderate with y =377 · x^0.282^ at 1.5T (R^2^ = 0.717), and y =406 · x^0.294^ at 3T (R^2^ = 0.715). Measures of reproducibility of T1 appeared superior to T2*.

**Conclusions:**

T1 mapping at 1.5T and at 3T can identify individuals with significant iron loading as defined by the current gold standard T2* at 1.5T. However, there is significant scatter between results which may reflect measurement error, but it is also possible that T1 interacts with T2*, or is differentially sensitive to aspects of iron chemistry or other biology. Hurdles to clinical implementation of T1 include the lack of calibration against human myocardial iron concentration, no demonstrated relation to cardiac outcomes, and variation in absolute T1 values between scanners, which makes inter-centre comparisons difficult. The relative merits of T1 at 3T versus T2* at 3T require further consideration.

## Background

Since a biological pathway for body iron surplus elimination is non-existent in humans, excess iron from gut absorption or transfusion is stored in a stable hemosiderin form but once this storage capacity is exhausted free iron can be left unbound. This free ferrous iron can consequently catalyse cellular Fenton reactions and create highly reactive hydroxyl radicals causing mitochondrial dysfunction, [1] apoptosis and other cellular dysfunction [2–4]. The most common cause of iron overload is the transfusion dependent anaemias, in which cardiac iron toxicity and contractile dysfunction can lead to heart failure, arrhythmias and death [5, 6].

Cardiovascular magnetic resonance (CMR) T2* assessment of myocardial iron burden at 1.5 Tesla (T) [7] plays a crucial role in the early diagnosis and management of patients with cardiac iron overload. [8] It has been validated against tissue iron concentration, [6, 9] and there is a powerful relation between low T2* values and future occurrence of heart failure [5]. It is used worldwide with T2* values between centres having excellent intercenter reproducibility, [10, 11] which allows consistency of clinical management between centres and a coherent approach to international guidelines [12]. Data also shows that adoption of T2* CMR leads to a substantial reduction in mortality in thalassaemia, which has been attributed to the adoption of aggressive iron chelation tailored to the heart before the onset of heart failure [13].

Against this background of substantial gains in clinical management of transfusion dependent patients over the last 15 years are new advances in hardware and software technology. Increasingly 3T scanners are entering clinical practice and these may offer advantages over conventional 1.5T magnets for iron burden quantification. Unfortunately, 3T CMR may suffer from increased artefacts making myocardial T2* assessment challenging, particularly in patients with a high degree of iron overload [14, 15]. Another more recent advance is improved T1 mapping of the heart by CMR. The Modified Look-Locker inversion recovery (MOLLI) and its shortened version (ShMOLLI) T1sequences (as well as a number of other sequence variants) have now been used to assess myocardial T1. Native T1 measurements have shown promise in the assessment of diffuse interstitial fibrosis, [16] and may help characterise myocardial infiltration in amyloidosis or Anderson-Fabry disease [17, 18]. T1 measurements can also be combined with gadolinium for quantification of extracellular myocardial volume, which is promising for clinical application for assessment of prognosis in heart disease, [19, 20] and the measurement of myocardial amyloid burden [21]. Native T1 has been shown to shorten with increasing tissue iron concentration in vitro, [22] and we recently reported the use of T1 in thalassaemia patients at 1.5T [23]. Therefore T1 is a credible technique to evaluate for iron measurement. We further investigated the relationship between native T1 at 1.5T and 3T against the current gold standard technique of BB T2* at 1.5T in normals and a spectrum of patients with transfusion dependent anaemia.

## Methods

### Patients and study design

A total of 53 consecutive subjects were prospectively recruited over a 6 month period from September 2012 to February 2013 from referrals for cardiac siderosis screening or follow-up. A wide dynamic range of iron loading was present in the patient group and was a realistic reflection of our population. Patients had successive CMR scans at both 1.5T and at 3T. A group of 20 matching healthy volunteers was also scanned. Exclusion criteria were: 1) claustrophobia, 2) metallic implants or permanent pacemaker and 3) inability to hold recumbent position for >15 minutes. The study protocol was approved by the local ethics committee, and all subjects gave written informed consent.

### Image acquisition

The BB T2* and MOLLI T1 CMR protocol was performed on a 1.5T Sonata scanner, and then a 3T Skyra scanner (both Siemens Medical Systems, Erlangen, Germany).

#### BB T2* acquisition

At 1.5T, a four-element cardiac phase-array coil was used. After routine localizer acquisitions, an ECG gated single breath hold multi-echo sequence was acquired at a single mid-ventricular short axis slice with a 10 mm thickness at eight separate echo times (2.6–16.74 ms, at 2.02 ms increments). The BB sequence used a flip angle of 35°, a matrix of 128 X 256 pixels a field of view (FOV) of 40 cm, repetition time of 20 ms between each radiofrequency (RF) pulse and a sampling bandwidth of 810 Hz per pixel. Furthermore, for the black blood preparation, double inversion pulses were applied at the R wave trigger and the inversion time (TI) was set to extend into diastole.

#### Native MOLLI T1 acquisition

The sequence used for myocardial T1 evaluation has been reported elsewhere [23]. In brief, non-selective inversion recovery (IR) prepared ECG-synchronized Look-Locker trains were performed in a 5 (3) 3 consecutive pattern within a single breath-hold. Each of the three trains started with a non-selective adiabatic inversion pulse that used inversion times of 100, 180 and 260 ms, after which multiple single-shot images were acquired in successive heartbeats. Therefore, a set of 11 sources images with different inversion times were obtained. Data acquisition consisted of a single mid-ventricular short axis slice balanced steady-state free precession (SSFP) sequence with acceleration factor 2. Bandwidth was 1085 Hz/pixel, base matrix of 256 pixels, flip angle 35°, FoV 400 mm, TR = 3.9 ms, TE = 1.12 ms, voxel size 2.1 x 1.4 x 10 mm. At 3T, a 3 (3) five sequence was acquired. The inversion times were 120, 200 and 280 ms. A set of 11 sources images with different inversion times were obtained. Data acquisition consisted of a single mid-ventricular short axis slice SSFP sequence with acceleration factor 2. Bandwidth was increased to 1395 Hz/pixel, base matrix of 256 pixels, flip angle 35°, FoV 400 mm, TR = 3.9 ms, TE = 1.14 ms, voxel size 2.1 x 1.4 x 10 mm. Motion correction was used on all sequences. T1 maps were generated by the scanner at 1.5T and 3T.

### T2* quantification

The myocardial T2* quantification with truncation has been described elsewhere [7]. In brief, dedicated software was used (Thalassaemia tools, a plug-in of CMRtools, Cardiovascular Imaging Solutions, London). The entire thickness of the cardiac interventricular septum was delineated to measure signal intensity (SI) decay at each echo time. The SI was subsequently plotted against echo time and a mono-exponential trendline was fitted to the decay curve to derive T2* according to this equation:

$$ SI=S{I_0}^{.}{e}^{\hbox{--} TE/T2*} $$

T2* was subsequently transformed into its reciprocal R2* according to the equation:

$$ T2*=1000/R2* $$

Curve fitting was performed with the truncation method [24]. The conventional T2* cut-off value for the presence of significant cardiac iron overload at 1.5T of 20 ms was used [7].

### Native T1 quantification

An identical mid-ventricular septum region of interest (ROI) was used for T1 quantification. Delineation of the epicardial and endocardial borders was done on the T1 maps. The interventricular septum was subsequently isolated for quantification. Careful consideration was given not to include blood-pool or artefacts in the septal ROI. T1 maps were automatically derived as described previously, [25–27] by fitting the measured signals to the following equation:

$$ \mathrm{S}\mathrm{I}\ \left(\mathrm{T}\mathrm{I}\right) = \mathrm{A} - \mathrm{B}.{\boldsymbol{e}}^{-\left(\mathrm{T}\mathrm{I}/\mathrm{T}1\right)} $$

Where SI is the signal intensity at a specific inversion time (TI), and T1 can be approximated:

$$ \mathrm{T}1 \approx \mathrm{T}1*\ \left(\mathrm{B}/\mathrm{A}\ \hbox{--}\ 1\right). $$

When required, T1 was subsequently transformed into its reciprocal R1 using a similar equation:

$$ T1=1000/R1 $$

No gadolinium contrast agent was administered. A single, blinded, experienced observer performed analysis for BB T2* at 1.5T, and MOLLI T1 at 1.5T and 3T.

### Reproducibility

A group of 20 patients was randomly selected for inter-observer, intra-observer and inter-study variability measurements. These patients had levels of myocardial siderosis that covered the entire range of the disease (from absent to severe iron overload). For intra-observer variability, the same investigator blindly reassessed myocardial T1 and T2* on 2 occasions a month apart. For inter-observer variability, two highly experienced investigators separately and blindly reported T1 and T2* for each patient. Finally, these 20 patients underwent a repeated cardiac T1 scan at 1.5T and at 3T one hour after completing the previous scan. The same investigator randomly and blindly re-evaluated T1 in these subjects to obtain inter-study variability.

### Statistical analysis

Data are expressed as median (interquartile range), except where stated as being the median and full range of values. Comparison of T2* and T1 values between normal volunteers and iron overload patients was performed with Mann–Whitney *U* test. Association between T2*, T1, R2* and R1 values at different field strengths were evaluated with a linear or non-linear regression when appropriate. Coefficients of variation (CoV) and intra-class correlation (ICC) with 95 % confidence interval (CI) analysis were conducted to assess inter- and intra-observer variability in addition to inter-study variability of T1 values. For ICC, an alpha two-way mixed model with absolute agreement analysis was used. Finally, Bland-Altman plots were generated to further describe the reproducibility of T1 measurements at 1.5T and at 3T [28]. A P-value <0.05 was considered significant. All analyses were done using SPSS software version 22.0.0, IBM, Chicago, Illinois, USA.

## Results

### Patient characteristics and comparison with healthy volunteers

**Fig. 1:**
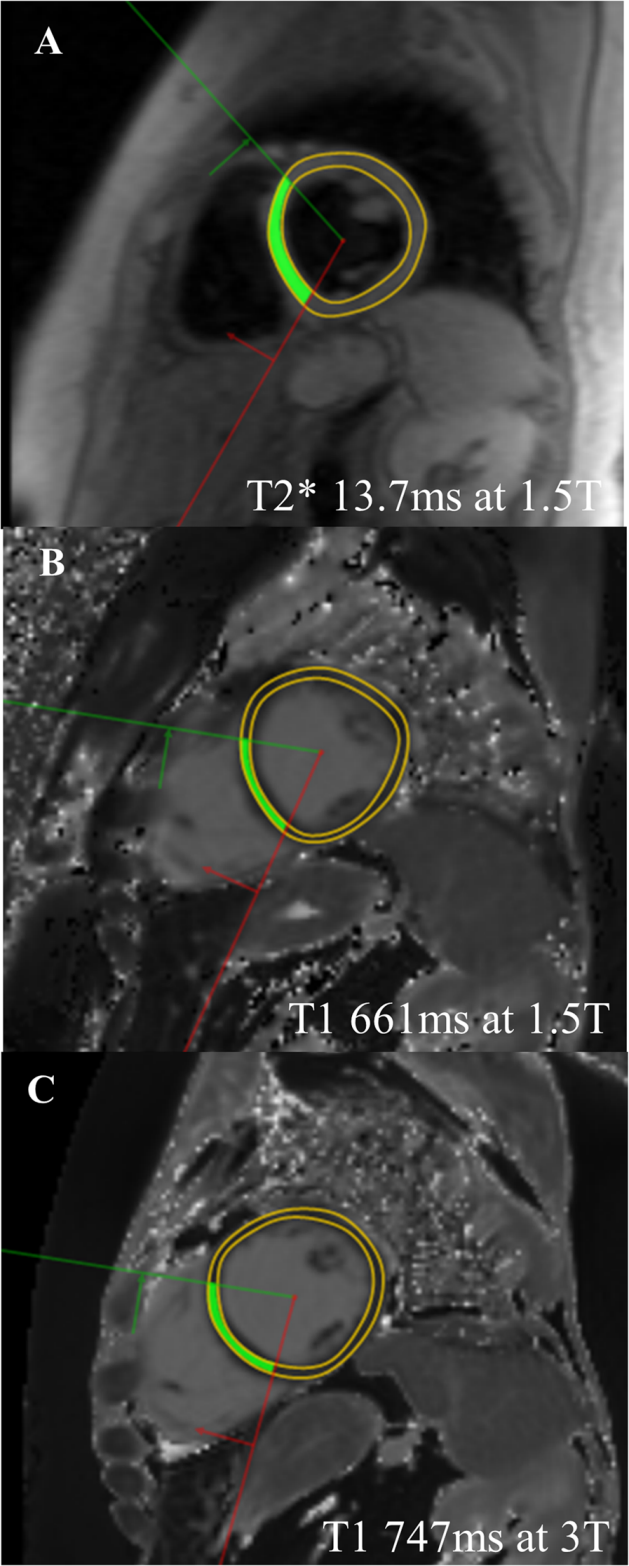
Example images from a single patient with iron overload: **a** Black blood T2* at 1.5T is reduced at 13.7 ms, indicating mild to moderate iron loading; **b** MOLLI T1 at 1.5T is reduced at 661 ms; **c** MOLLI T1 at 3T is longer than at 1.5T but still reduced at 747 ms

A cohort of 53 iron overload patients and 20 healthy volunteers were included. Their demographics and clinical characteristics are shown in Table 1 and typical images from a single patient are shown in Fig. 1. The median age of the patients was 35 (24–59) years old. The largest proportion of patients had β-thalassaemia (n = 22, 42 %). Median left ventricular ejection fraction by CMR was 64 % (59–67). Four patients did not undergo T1 quantification at 1.5T because of software issues on the scanner. In two of the patients with severe cardiac iron overload, T1 at 1.5T was not available. All patients underwent T1 quantification at 3T.

**Table 1:** Patient characteristics

Variables	Patients *N* = 53	Controls *N* = 20	*P*-value
Age (years)	35 [24–59]	34 [25–41]	0.45
Gender (Male/Female)	33/20	9/11	0.20
Diagnosis			
TM	22		
SCA	9		
Rare hemoglobinopathies	15		
MDS	4		
Other	3		
LVEF (%)	64 [59–67]		
Myocardial T2* (ms) at 1.5T	28.1 [20.4–34.4]	30.8 [29.0–34.4]	0.14
Full range	6.41–41.5	22.6–40.4	
Myocardial T1 (ms) at 1.5T	978 [865–1039]	1014 [974–1033]	0.21
Full range	661–1084	939–1059	
Myocardial T1 (ms) at 3T	1103 [927–1148]	1165 [1119–1194]	0.001
Full range	653–1222	1056–1224	

Data are expressed as median [interquartile range]. *LVEF* left ventricular ejection fraction, *MDS*, myelodysplasic syndrome, *SCA* sickle cell anemia, *TM* thalassemia major

In the normal volunteers control group, the median T1 at 1.5T was 1014 ms [974–1033] and the full range of values was 939–1059 ms. In accord with previous intellectual considerations defining the lower limit of normal of T2* to represent the onset of significant iron loading, so that low values should rarely be seen in the normal population, we used the lowest T1 observed in normals of 939 ms at 1.5T as the lower limit of normality for the scanner and sequence that was used in this report. The median T1 at 3T was longer at 1165 ms [1119–1194] and the full range of values was 1056–1224 ms. Using the same argument as above, we therefore set the lower limit of normal for significant iron loading as being the lower limit of the normal range at 3T which was 1056 ms.

### Association between T1 and T2* both at 1.5T

**Fig. 2:**
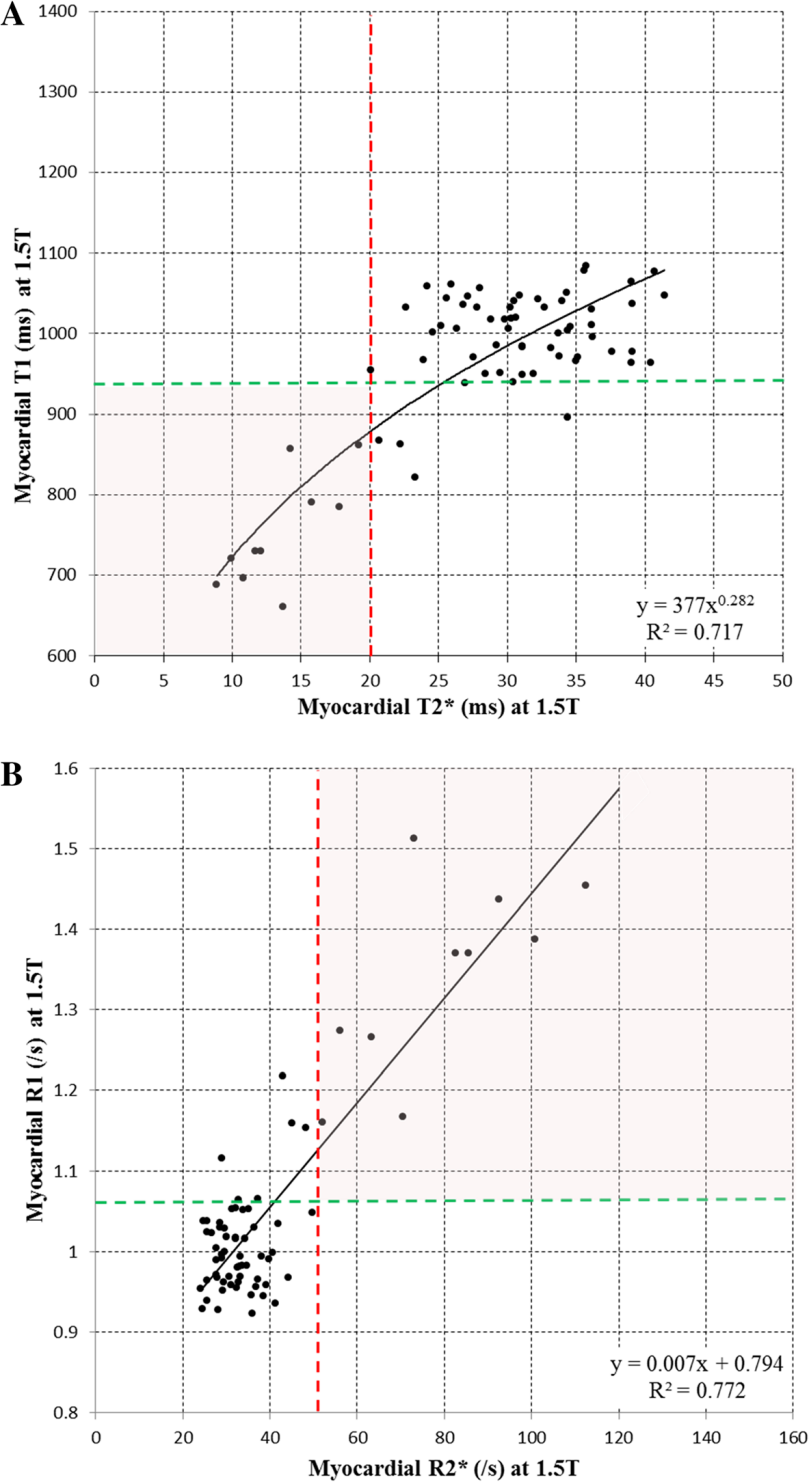
**a** Association between T1 at 1.5T and T2* at 1.5T; **b** Association between cardiac R1 at 1.5T and R2* at 1.5T

The relation between T1 and T2* at 1.5T is shown in Fig. 2a. A power regression model was fitted to the data yielding an equation of y = 377 · x^0.282^ (R^2^ = 0.717). All patients with cardiac iron overload (T2* <20 ms at 1.5T) had T1 values at 1.5T below the lower limit of normal T1 values in the control cohort (939 ms). Only four patients had T2* values >20 ms at 1.5T and had T1 values at 1.5T below the lower limit of T1 values found in the present normal cohort. As shown in Fig. 2b, the relation between R1 and R2* at 1.5T was linear (y = 0.007 · x + 0.794; R^2^ = 0.772).

### Association between T1 at 3T and T2* at 1.5T

**Fig. 3:**
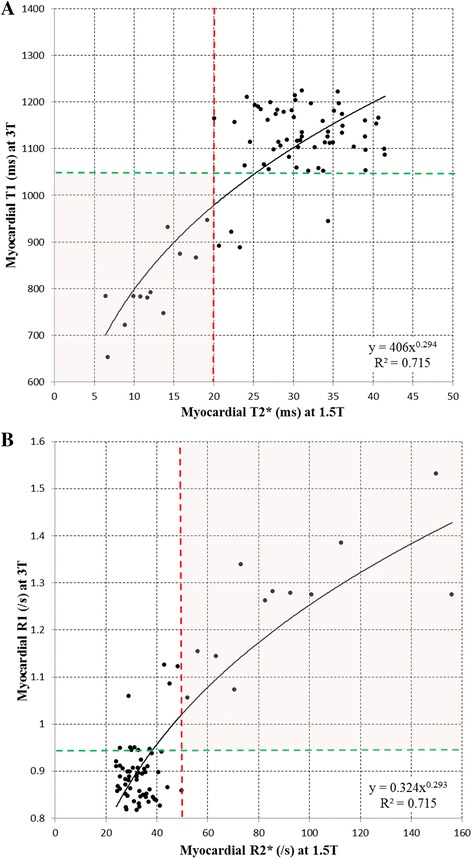
**a** Association between T1 at 3T and T2* at 1.5T; **b** Association between R1 at 3T and T2* at 1.5T

The relation between T1 at 3T and T2* at 1.5T is shown in Fig. 3a, and described by the power model regression with fit y = 406 · x^0.294^ (R^2^ = 0.715). All patients with a significant degree of iron overload (T2* <20 ms at 1.5T) had a T1 values at 3T <1056 ms (lower threshold of the normal volunteers). A total of seven patients with normal T2* values at 1.5T had T1 values below 1056 ms at 3T. The association between R1 at 3T and R2* at 1.5T is shown in Fig. 3b. The power regression equation for this relation was y = 0.324 · x^0.293^ (R^2^ = 0.715).

### Reproducibility

**Fig. 4:**
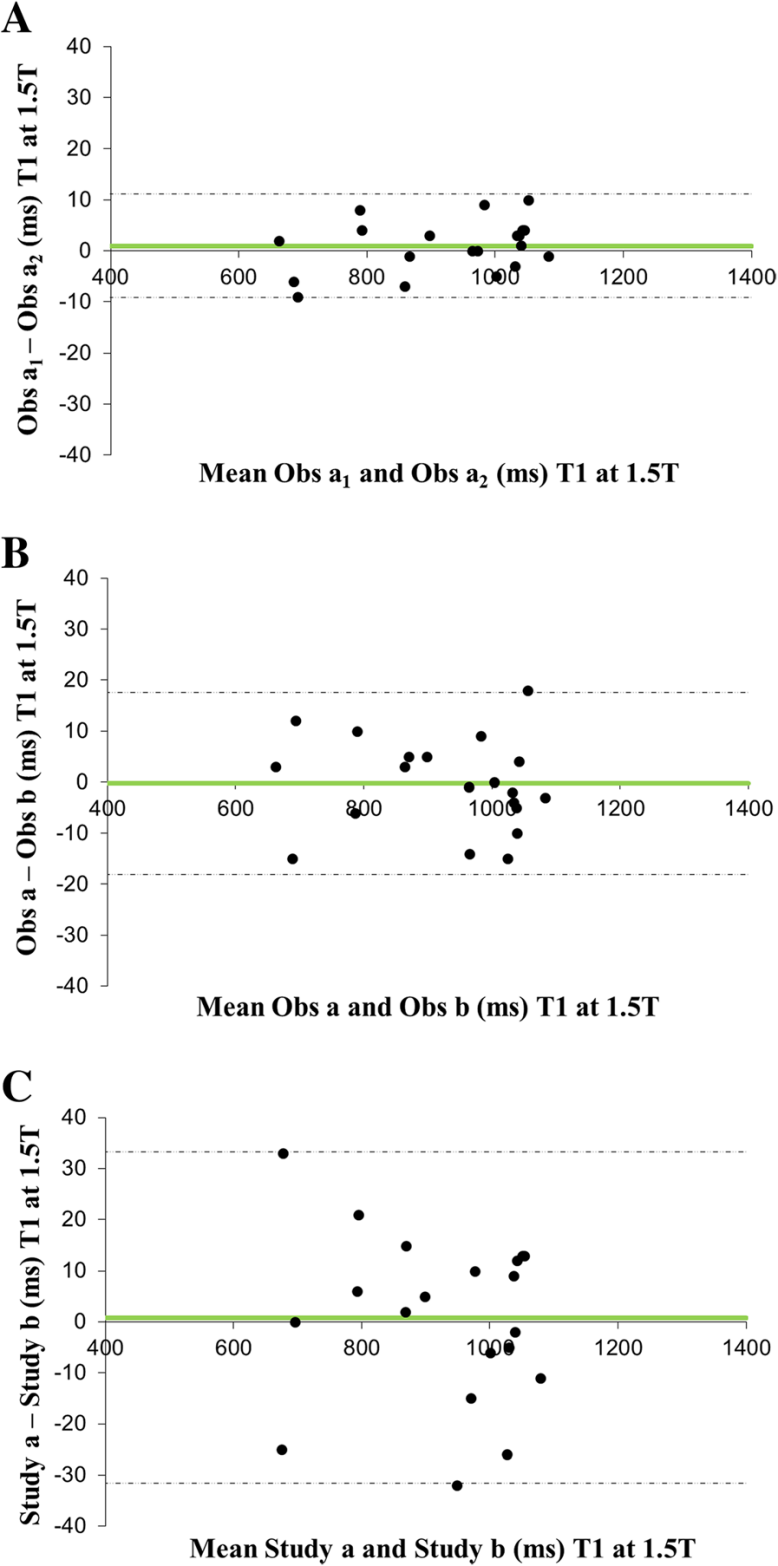
Bland Altman plots for T1 reproducibility at 1.5T **a** intra-observer; **b** inter-observer; **c** inter-study reproducibility

**Fig. 5:**
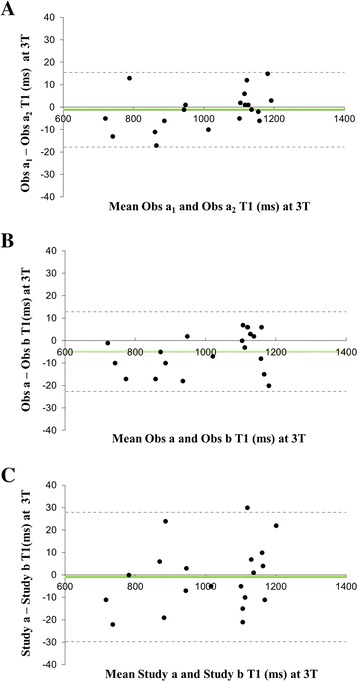
Bland Altman plot for T1 reproducibility at 3T **a** intra-observer; **b** inter-observer; **c** inter-study reproducibility

The intra-observer, inter-observer and inter-study variability of T1 at 1.5T and at 3T is shown in Table 2. There was excellent agreement between the repeated measurements of T1 at both 1.5 and 3T, but the most important measure of inter-study variability of T1 was better at 3T than 1.5T. The Bland-Altman plots confirm that there was no systemic bias in reproducibility of T1 at 1.5T (Fig. 4) and at 3T (Fig. 5). The T1 reproducibility for all measures at both 1.5T and 3T appeared superior to T2* at 1.5T.

**Table 2:** Reproducibility of myocardial T1 at 1.5T and 3T, and T2*at 1.5T

Myocardial T1		1.5T	3T
Intraobserver	CoV (%)	0.56	0.84
	ICC (95 % CI)	0.999 (0.998–1.000)	0.999 (0.997–0.999)
Interobserver	CoV (%)	0.98	0.89
	ICC (95 % CI)	0.998 (0.995–0.999)	0.996 (0.988–0.999)
Interstudy	CoV (%)	1.79	1.45
	ICC (95 % CI)	0.993 (0.982–0.997)	0.996 (0.989–0.998)
Myocardial T2*			
Intraobserver	CoV (%)	1.54	
	ICC (95 % CI)	1.000 (1.000–1.000)	
Interobserver	CoV (%)	2.76	
	ICC (95 % CI)	0.999 (0.999–1.000)	
Interstudy	CoV (%)	4.55	
	ICC (95 % CI)	0.994 (0.985–0.998)	

*Abbreviations: CI* confidence interval, *CoV* coefficients of variation, *ICC* intraclass correlation coefficient

## Discussion

These data show reasonable correlations between cardiac T1 and cardiac T2* measures across a wide range of cardiac iron loading, but with significant scatter, and there is reasonable agreement with our previous data comparing T1 and T2* at 1.5T in thalassaemia patients, [23] and with the more recent data from Sado et al., [29] although with differences as discussed below. There is a prima facie case therefore for supporting the notion that T1 has a potential role in iron measurement. The question that arises is what might be the relative advantages and disadvantages for using T1.

The advantages of T1 CMR include: A) At low iron levels, T1 is less affected by non-iron influences that affect T2* measurements, making T1 appear to be more suitable for clinical conditions where low iron levels may be responsible for disease pathogenesis; B) T1 has good inter-study reproducibility, which appears superior to T2* which may be of benefit for longitudinal patient follow-up and clinical trials. Counterbalancing views to these advantages are: A) The dominant pathologies associated with iron dysfunction by prevalence are those involving substantial iron overload (eg transfusion dependent anaemias, haemochromatosis) and for these conditions there is rather little obvious merit in defining iron levels moving within the non-significant range, when movements of T2* are robustly identifiable in the low-risk mild to moderate iron overload range of cardiac T2* between 10–20 ms. Nonetheless, some more unusual pathologies are apparently associated with iron dysfunction at low iron levels, [23, 30] for which T1 might be a more suitable iron assay than T2*. B) The improved inter-study reproducibility of T1 measurements is potentially valuable. This being said, it is clear that the performance of cardiac T2* could hardly be described as weak. The relative CoV’s at 1.5T for T1 and T2* in this paper were 1.79 % vs 4.55 % with near identical ICCs of 0.993 vs 0.994. Both parameters therefore appear to be strong performers in absolute terms. Certainly sample sizes in the order of 60 have been used in randomised controlled trials using T2* as the primary endpoint, that showed a significant difference between the performance of competing iron chelators, [31, 32] and a sample size of less than this may not be clinically credible. However, T1 might have an advantage if 2 drugs with similar efficacy were to be compared. This would depend on other factors as well, including standardisation of T1 measurements, as addressed further below. It should be noted that improvements in T2* technology may improve reproducibility [33].

It has also been suggested that cardiac T1 might have improved utility for identifying patients with cardiac iron loading through a better delineation of the lower limit of normal for cardiac iron loading [29]. It is difficult to sympathise with this view. The normal range for cardiac iron has received rather little scientific attention from basic science, with the most commonly cited paper from Collins and Taylor published in 1987 indicating a normal range in eight hearts of 0.34 mg/g dw (range 0.29–0.47) [34]. From the work of Carpenter et al., [6] the accepted mean normal myocardial T2* value of 40 ms yields a myocardial iron concentration of 0.5 mg/g dw which is in reasonable accord with this upper range. It is remarkable that such a level of agreement should occur given that the methodologies of myocardial iron measurement are completely different and performed some 24 years apart, and does lend credence to the T2* calibration. The definition of the normal range of cardiac iron from T2* is however philosophically challenging. Early attempts to justify the lower limit of normal were made on the simple basis of taking the mean measurement in normals and subtracting two standard deviations. However, it is now known that T2* is not normally distributed and this approach which leaves the lower ~5 % of the normal population with “cardiac iron overload” is neither sensible statistically nor satisfying clinically. A more pragmatic approach is to sample a normal population and take the lowest T2* value obtained as the lower threshold (assuming that the normal population does not include a subject with occult iron loading). For myocardial T2*, this has generally been accepted as 20 ms, although other values can and have been suggested. A further persuasive argument for definition of a normal range is to associate the T2* to outcomes, because this is patient focussed rather than test focussed. The work from Kirk et al. clearly showed that heart failure occurs in a very high proportion of cases only when myocardial T2* <10 ms (myocardial iron equivalent of 2.7 mg/g dw). This threshold of severe iron loading is therefore beyond question. However, it is clear that to suggest that there needs a hard and fast rule to define the threshold for the onset of mild cardiac iron overload is somewhat misguided and unhelpful. Since the range of myocardial T2* between 20 ms and 10 ms is large and easily measured, it serves as a valuable early warning zone that problems are likely in the future should the T2* drop further. Whether this range starts at 20 ms or at a slightly higher value takes no account of the continuous nature of iron loading and suggests a vital clinical importance for decision making based on an arbitrary threshold, which is not the case. It is therefore possible to consider that questions of whether T1 or T2* better identify a threshold for early cardiac iron loading are moot. The position that T1 is better for identifying the lower limit of normal for myocardial T1 is also problematic unless the measurement can be standardised, with for example Sado et al. reporting a value of 904 ms (Sado et al. [29]) vs 939 ms in this report.

Another potential advantage of T1 measurement of iron concentration which is identified in this paper, is the use of T1 at 3T. There has been substantial increase in the use of 3T for MR of the brain and musculoskeletal system, and a number of centres no longer have 1.5T scanners available causing problems for thalassaemia patients. The two most likely solutions to this problem are to calibrate T2* at 3T against 1.5T, on which progress has already been made, [14, 15, 35] or to consider using T2 or T1 mapping. In this paper, we show that T1 mapping at 3T is feasible and causes only rather modest prolongation of T1 values (median normal at 1.5T vs 3T: 1014 ms vs 1165 ms). The scatter of T1 vs T2* values at 3T (Fig. 3a) is similar to that at 1.5T (Fig. 2a) and the inter-study reproducibility of T1 at 3T (1.45 %) was slightly superior to 1.5T (1.79 %). This approach may be worth exploring further, because T2* imaging at 3T has increased artefacts and shorter T2* values, which can be problematic to image compared with imaging at 1.5T.

A problem associated with the current implementation of T1 mapping is the variation of absolute values of T1 between sequences and scanners. This is illustrated in this paper where the median value of T1 at 1.5T in normal subjects was 1014 ms which compares with the mean of 968 ms from Sado et al. [29] Sado used the shortened form of MOLLI (shMOLLI) as well as MOLLI and they found a 25 ms difference between their techniques [29]. This difference is not insignificant, being almost one standard deviation in their normal range (32 ms). These differences arise from the complex nature of measuring T1, which is affected by which sequence is used, [36] and a large number of technical parameters that are difficult to standardise [27]. Work is ongoing to try to resolve these issues, [37, 38] but T1 mapping for iron assessment would currently require each centre to establish its own normal range. This issue for T1 is less problematic for marked iron loading however, because the dynamic range of decrement in T1 reaches ~300 ms, although the direct comparison of T1 values between centres would remain difficult. It is also clear that the direct comparison of T1 against T2* values which are understood clinically, relies considerably on the modelling used to relate these 2 parameters, and this is currently unresolved. The three papers that have modelled T1 against T2* at 1.5T have found significantly different regression lines due to data scatter, such that for example, a T2* of 10 ms approximates to a T1 of 620 ms by Feng et al., [23] 650 ms by Sado et al., [29] and 720 ms in the current paper. This situation is different to that faced by T2*, which proved straightforward to transfer between centres, [10] between different manufacturers’ scanners, [39, 40] and between centres internationally, [11, 39] yielding excellent intercenter agreements of T2* values at an early stage of development [11, 39].

Some further technical issues need to be considered in the use of T1 for iron assessment. Diffuse fibrosis raises native T1, whereas myocardial iron lowers native T1. There is debate about the prevalence of myocardial fibrosis in thalassaemia, [41, 42] but its occurrence would increase the T1 value causing iron underestimation. T2* does not appear to be significantly affected by myocardial fibrosis [43, 44]. A further confounding issue for T1 measurements is that with increasing iron loading, T2* effects influence the measurement of T1, [16, 23] which affects its accuracy. T1 measurements need to be performed on carefully selected regions of interest in the myocardium that exclude other tissues with very different T1, such as blood and fat, to prevent erroneous T1 values [45]. Finally, there is a report that T1 varies by gender and age, with increased values by 24 ms in females up to the age of 45 years, [45] which would be unwelcome if substantiated. All these issues need further consideration.

Further issues with the validation of T1 also exist, that are troublesome. Clearly this paper and others have shown that T1 can be used to measure tissue iron concentration, but in humans the only comprehensive validation in the heart has been performed using T2*. Myocardial T2* measured at 1.5T is firmly established as the gold standard measurement of myocardial iron concentration, [12] and this is because of a number of important reasons: 1) The scan is fast (short single breath-hold) which makes it easy for patients to tolerate (even children) and therefore efficient on time use of the MR scanner; 2) It is robust in clinical practice and scan failures are unusual; 3) Absolute T2* values are highly reproducible between different manufacturers scanners and sequences allowing individual centres to directly compare patients’ results; [39] 4) The inter-study reproducibility is very good allowing small sample sizes for clinical treatment studies in the order of 60 patients; 5) The T2* technique is calibrated directly to human heart tissue, [6, 9] but attempts to calibrate T1 against human heart tissue have to date failed, [46] as a result of the effects of formalin on T1 relaxation. 6) The relation between cardiac T2* value and future cardiac events is well described and powerful; [5] 7) The technique is widely disseminated in clinical practice around the world, [47] which is not the case for T1; 8) And a reduction in cardiac mortality has been demonstrated using T2* as a guide to aggressive iron chelation therapy that is tailored to the heart in advance of the onset of heart failure [13]. Therefore any MR scanning alternative to T2* has a substantial burden of re-validation and outcomes analysis to climb to reach the same level of clinical confidence in use. Another approach to this problem is simply to assume that putative alternatives such as T1 would simply be calibrated against conventional T2* values at 1.5T and therefore directly cross-referenced to the established cardiac T2* literature. Such an approach has merit, but certainly leaves room for doubt as to veracity of the measurement, because there is no guarantee that T1 and T2* measure identical chemical iron species with identical effects on outcome. The additional problem already mentioned is that there are significant problems in identifying the best fit curve between T1 and T2* which leads to uncertainty.

## Conclusions

Native T1 mapping at 1.5T appears to be promising as a tool to evaluate myocardial iron. However, considerable further work is required in standardisation and transferability between scanners and centres, as well as in understanding whether T1 can be used as a substitute for T2* in the absence of direct studies of calibration and linkage with cardiovascular outcomes. The established practice worldwide of using T2* at 1.5T is unlikely to change in the near future without persuasive additional arguments. However, T1 measurements at 3T may prove useful where T2* measurements can be problematic.

## Abbreviations

BB: Black blood
CI: Confidence interval
CMR: Cardiovascular magnetic resonance
CoV: Coefficient of variance
ECG: Electrocardiogram
FOV: Field of view
MOLLI: Modified Look-Locker Inversion recovery
ICC: Intraclass correlation coefficient
IR: Inversion recovery
RF: Radiofrequency
ROI: Region of interest
shMOLLI: Shortened Modified Look-Locker Inversion recovery
SI: Signal intensity
SSFP: Steady State Free Precession
T: Tesla
TI: Inversion time
